# Hematology Laboratory Survey

**DOI:** 10.4274/tjh.2018.0273

**Published:** 2018-11-13

**Authors:** Rujittika Mungmunpuntipantip, Viroj Wiwanitkit

**Affiliations:** 1 26 Medical Center, Clinic of Hematology, Bangkok, Thailand; 2 Dr. DY Patil University, Pune, India

**Keywords:** Hematology, Laboratory, Survey

## To the Editor,

We read the publication “Results of the Hematology Laboratory Survey: What Has Changed in Eight Years?” with great interest. Kozanoğlu et al. [1] noted that “Hematology laboratories have not been defined in the Turkish Medical Laboratories Regulation (2010, 2013), which regulates procedures and principles regarding the planning, licensing, opening, regulating, classifying, monitoring, controlling, and terminating of activities of medical laboratories”. We would like to share ideas from our country in Indochina. In Thailand, there is no isolated hematology laboratory. All clinical hematology investigation is performed by a standard clinical laboratory medicine center [2]. Routine laboratory survey and quality surveillance for accreditation is the basic requirement. This might be a good way for quality control of laboratory processes. Indeed, there is a need for clinical pathologists or clinical hematologists for the management of a hematology laboratory. This is necessary for the assurance of the quality of laboratory diagnoses. The concept of isolated hematology and combination with other clinical laboratories in a single laboratory medicine unit is an interesting topic for further discussion of the advantages and disadvantages.
