# Supplementary material for: Hematology Laboratory Survey
Source: Turk J Haematol. 2018 Nov 13;35(4):303–4. doi: 10.4274/tjh.2018.0273 (PMC6256822; doi:10.4274/tjh.2018.0273)
Supplement: Related texts

# Reply to the Authors

**Authors:** İlknur Kozanoğlu, Türkan Patıroğlu, Klara Dalva, Gülderen Yanıkkaya Demirel, Teoman Soysal, Muzaffer Demir

## To the Editor,

We read the letter from Mungmunpuntipantip and Wiwanitkit regarding our publication on “Results of the Hematology Laboratory Survey: What Has Changed in Eight Years?”

First of all, we would like to thank the authors, that they have shared their experiences and ideas from their country. We understood that all clinical hematology investigation was performed by a standard clinical laboratory medicine center in Indochina and also, there is no isolated hematology laboratory in their country.

We all agree that the concept of isolated hematology and combination with other clinical laboratories in a single laboratory medicine unit is an interesting topic for further discussion on advantages and disadvantages. Many hematology laboratories have closed and/or were included into a central laboratory system after starting new regulations in Turkey. The authors also agree with us that there is a need for the clinical hematologist for management of the hematology laboratory. However, after the new regulations in our country, the managers of hematology laboratories became biochemistry specialists. But it is very important to evaluate specific tests in the hematology laboratory and to integrate them with the clinical status of patients. Therefore, the removal of clinical hematologists from hematology laboratories was a significant disadvantage. The routine laboratory survey and quality surveillance for accreditation is the basic requirement not only for hematology laboratories but also in all laboratories. However, after the new regulations implemented, only the quality issues will not be sufficient to solve problems related to hematology laboratories in Turkey.

**Best Regards,**
